# Differences in style length confer prezygotic isolation between two dioecious species of *Silene* in sympatry

**DOI:** 10.1002/ece3.1350

**Published:** 2015-06-19

**Authors:** Phil Nista, Amanda N Brothers, Lynda F Delph

**Affiliations:** Department of Biology, Indiana UniversityBloomington, Indiana, 47405

**Keywords:** Hybridization, pollination, reinforcement, reproductive character displacement, reproductive isolation

## Abstract

One fundamental signature of reinforcement is elevated prezygotic reproductive isolation between related species in sympatry relative to allopatry. However, this alone is inadequate evidence for reinforcement, as traits conferring reproductive isolation can occur as a by-product of other forces. We conducted crosses between *Silene latifolia* and *S. diclinis*, two closely related dioecious flowering plant species. Crosses with *S. latifolia* mothers from sympatry exhibited lower seed set than mothers from five allopatric populations when *S. diclinis* was the father. However, two other allopatric populations also exhibited low seed set. A significant interaction between style length and sire species revealed that seed set declined as style length increased when interspecific, but not intraspecific, fathers where used. Moreover, by varying the distance pollen tubes had to traverse, we found interspecific pollen placement close to the ovary resulted in seed set in both long- and short-styled *S. latifolia* mothers. Our results reveal that the long styles of *S. latifolia* in sympatry with *S. diclinis* contribute to the prevention of hybrid formation. We argue that forces other than reinforcing selection are likely to be responsible for the differences in style length seen in sympatry.

## Introduction

When two lineages evolve in allopatry, drift and selection can lead to intrinsic incompatibilities and result in speciation (Dobzhansky 1937). However, if these species are brought into secondary contact following this time of divergence, gene flow between them can still occur if reproductive isolation is not complete. At this point, natural selection can increase prezygotic gene flow barriers to reinforce their isolation, if hybrid offspring have lower fitness than pure-species offspring (Fisher 1930; Dobzhansky 1940). This phenomenon is known as reinforcement (Blair 1955), which can lead to reproductive character displacement (Howard 1993; Lukhtanov et al. 2005). If reinforcement has occurred, then populations of species in sympatry should show stronger reproductive isolation than populations in allopatry (Grant 1966, 1971), or, in the words of Dobzhansky, reinforcement should occur “where the danger of hybridization between the species is greatest” (Dobzhansky 1951, p. 209). Furthermore, it has been argued that reinforcing selection should act mainly on traits that prevent interspecific matings (e.g., either premating- or postmating-prezygotic isolation), leading to empirical studies specifically investigating whether prezygotic isolation is strongest in sympatry (Coyne and Orr 2004).

In addition to evidence from numerous studies with animals (Marshall et al. 2002; Coyne and Orr 2004), evidence consistent with reinforcement has been observed in flowering plants (see reviews in Kay and Schemske 2008 and Hopkins 2013). Some of the earliest as well as the most complete evidence for reinforcement is from studies on *Phlox drummondii*. This species exhibits character displacement in petal color when in sympatry with *Phlox cuspidata*, which reduces hybridization between these two species (Levin 1985). More recently, restricted gene flow and drift were ruled out as being responsible for the character displacement, in a study showing that the gene responsible for one color component had been selected to differ in sympatry (Hopkins et al. 2012). In addition, results from field experiments ruled out the alternative possibility of the character displacement being caused by local adaptation to the physical environment, and again showed that color had a strong effect on pollinator movement and hybridization (Hopkins and Rausher 2012). Moreover, similar character displacement of petal color between sympatric species occurs in at least one additional pair of sympatric *Phlox* (Levin and Kerster 1967). Together, these studies strongly support the selection for petal color of pairs of sympatric *Phlox* species to differ, as a way of reinforcing prepollination reproductive isolation between them. Similar prepollination reproductive isolation via character displacement of flower color has also been shown in a Solanaceae clade. Muchhala et al. (2014) showed that competition for hummingbird pollinators appears to have led to petal-color diversification in sympatry as a way of decreasing the movement of pollinators among species, and thereby limiting the formation of unfit hybrids.

In contrast, the perennial, hummingbird-pollinated herbs, *Costus pulverulentus* and *C. scaber*, also exhibit an increase in reproductive isolation in sympatry, but the mechanism occurs postpollination (Kay and Schemske 2008). Pollen is transferred from *C. pulverulentus* to *C. scaber* by naturally occurring pollinators (Kay 2006), yet no hybrids occur. When pollinations between these two species were performed by hand in a greenhouse using allopatric and sympatric populations, seed set was lowest when pollination was between sympatric individuals as a consequence of pollen–pistil interactions (Kay and Schemske 2008). A larger crossing experiment in the same genus revealed a similar pattern in another sympatric species pair. These crosses with *Costus* suggest that the selection for reproductive isolation can produce postpollination-prezygotic barriers to gene flow. This can be easily reconciled with the previously discussed results from *Phlox,* because both “prepollination-” and “postpollination-prezygotic” barriers to gene flow are consistent with Mayr (1963) and Dobzhansky’s (1970) concept of prezygotic reproductive isolation (Harrison 2012). That is, the selection is still acting to prevent investment in unfit hybrid offspring.

Additional case studies are required to address what types of traits might have experienced reinforcement in plants (Hopkins 2013) and at what stages such reinforcement occurs (Moyle et al. 2004). While there are obvious advantages to reinforcement prior to pollination, it is not clear whether reinforcement most often produces conspicuous, prezygotic barriers as in *Phlox* and Solanaceae*,* or cryptic, postpollination-prezygotic barriers as in *Costus*. Moreover, while reinforcement is one mechanism that can lead to reproductive character displacement in sympatry, other mechanisms are possible, including divergent selection in allopatry leading to differences that prevent fusion when brought into sympatry (known as “differential fusion”). In addition, local adaptation can lead to ecological character displacement in sympatry (Hoskin and Higgie 2010; Hopkins 2013).

Here, we examine how postpollination-prezygotic processes contribute to reproductive isolation of two flowering plant species, *Silene latifolia* and *S. diclinis*, two closely related dioecious species with sex chromosomes. Previous work with *S. latifolia* and *S. diclinis* suggests that several criteria necessary for reinforcement, or its potential end product, reproductive character displacement (see Howard 1993), are met in this pair. These two species diverged from a common ancestor around 1.26 Myr ago and are thought to have evolved in allopatry based on a multigenic analysis of gene flow (Muir et al. 2012). Although they now occur in sympatry (Prentice 1976; Brothers and Atwell 2014), hybrids between these species do not occur in nature, even though they overlap in flowering time in the area of sympatry (A. Brothers and L. Delph, pers. obs.). Prentice (1978) found extremely low viable seed production from interspecific crosses between this species pair and suggested that reinforcement may have added to the reproductive isolation between them. However, other crossing studies between these two species using an allopatric population of *S. latifolia* produced numerous viable hybrid seed (Brothers and Delph 2010; Demuth et al. 2014). The production of these hybrid seeds was shown to be costly, because of the existence of postzygotic reproductive incompatibilities that were consistent with Haldane’s rule (Brothers and Delph 2010). As is commonly found (Turelli and Moyle 2007), compliance with Haldane’s rule was asymmetrical in crosses between this species pair; hybrid *F*_1_ males were only rare and suffered the most in terms of sterility when *S. latifolia* was the mother. This is likely to lead to asymmetry in terms of the costliness of interspecific mating, potentially setting up stronger selection in sympatry to avoid matings in which *S. latifolia* is the mother and *S. diclinis* is the father (Yukilevich 2012).

In addition, reinforcing selection is more likely to be strong, or have been strong in the past (Pfennig 2003), if there is or was something to reinforce. In other words, gene flow via mating between species must occur (or have occurred), but it must not occur so often as to lead to homogenization (Grant 1994; Servedio and Kirkpatrick 1997; Kirkpatrick 2000; Nosil et al. 2003; Coyne and Orr 2004). For plants, this means that reinforcement will be more likely if pollinators occasionally visit both species, but usually discriminate between them (Grant 1994). Flowers of the species studied here open at different times (dusk vs. morning) and are primarily pollinated by different insects (moths vs. bees; Brothers and Atwell 2014). Although pollinating moths visit only *S. latifolia* in the area of sympatry, diurnal visitors to *S. diclinis* are less discerning, and will visit *S. latifolia* flowers that have remained open into the early morning (Brothers and Atwell 2014). Hence, pollen is likely to flow between the two species in this manner in the area of sympatry.

We determined whether postmating-prezygotic isolation between *S. latifolia* and *S. diclinis* differed between sympatry vs. allopatry. We particularly focused on style length. This focus was based on studies that have shown that style-length differences among species often lead to asymmetric crossing success (Buchholz et al. 1935; Smith 1970; Carney and Arnold 1997; Diaz and Macnair 1999; Tiffin et al. 2001; Kay and Schemske 2008; Lee et al. 2008). Moreover, style length generally varies with flower size, and flower size has been shown to exhibit character displacement among sympatric species of *Mimulus* (Grossenbacher and Whittall 2011). This suggests that reinforcing selection may promote divergence of flower size in regions of sympatry. However, the selection on flower size (and hence style length) has been shown to be imposed by a variety of biotic and abiotic factors, including pollinators (Sandring and Ågren 2009; Parachnowitsch and Kessler 2010; Dudash et al. 2011), herbivores (Galen and Cuba 2001), and water availability (Galen 2000; Mojica and Kelly 2010). As a consequence, even though style length might differ in sympatry, reinforcement is only one possible underlying cause. Nevertheless, if style-length differences exist and contribute to reproductive isolation only in sympatry, this would be congruent with reinforcing selection having led to character displacement, and further investigation into this phenomenon would be warranted.

We performed experimental hand pollinations on plants growing in the greenhouse, followed by comparisons of seed set among different cross types, including crosses between individuals from the area of sympatry and individuals from several allopatric populations that varied in style length. We also manipulated the placement of *S. diclinis* pollen on the styles of S. *latifolia* females. Because receptive papillae are present along the entire length of the style, from its tip to its base, one can vary the distance that pollen has to travel to reach an ovule by placing pollen at the tip (away from the ovary) or the base (near to the ovary) of the style. We therefore performed tip and base pollinations on flowers of *S. latifolia* females from the long-styled sympatric population as well as females from one short-styled allopatric population. If factors other than style length contribute to the low interspecific seed set in sympatry when *S. latifolia* is the mother, then altering the distance that the pollen from *S. diclinis* has to travel might not affect seed set. In contrast, seed set should differ more for tip vs. base pollinations in females from the long-styled population compared to the short-styled population if style length were the factor determining whether reproductive isolation (i.e., lack of fertilization) occurred.

## Methods

### Study system and growth conditions

*Silene latifolia* is a weedy, herbaceous perennial that naturally occurs across most of Europe (Baker 1948), having spread from Iberian and Balkan refugia postglaciation (Taylor and Keller 2007). Its large, white flowers are borne on upright shoots, open at dusk, and are pollinated primarily by the nocturnal moth *Hadena bicruris* in its native range (Brantjes 1976; Jurgens et al. 1996). In contrast, *Silene diclinis* is an endangered, very narrowly distributed endemic, occurring naturally in only a 9 km by 18-km region of southeastern Spain near Xativa (Montesinos et al. 2006), with local subpopulations that are not genetically differentiated from each other (Prentice 1984; Harper 2010). Effectively, only one population of *S. diclinis* exists. The smaller, pink flowers of *S. diclinis* are borne on prostrate branches, open during the day, and are pollinated by bees and other diurnal floral visitors (Prentice 1976; Brothers and Atwell 2014). Previous reports indicate that these two *Silene* species exhibit both premating-prezygotic (Prentice 1978; Brothers and Atwell 2014) and postzygotic (Brothers and Delph 2010) reproductive isolation.

**Figure 1:**
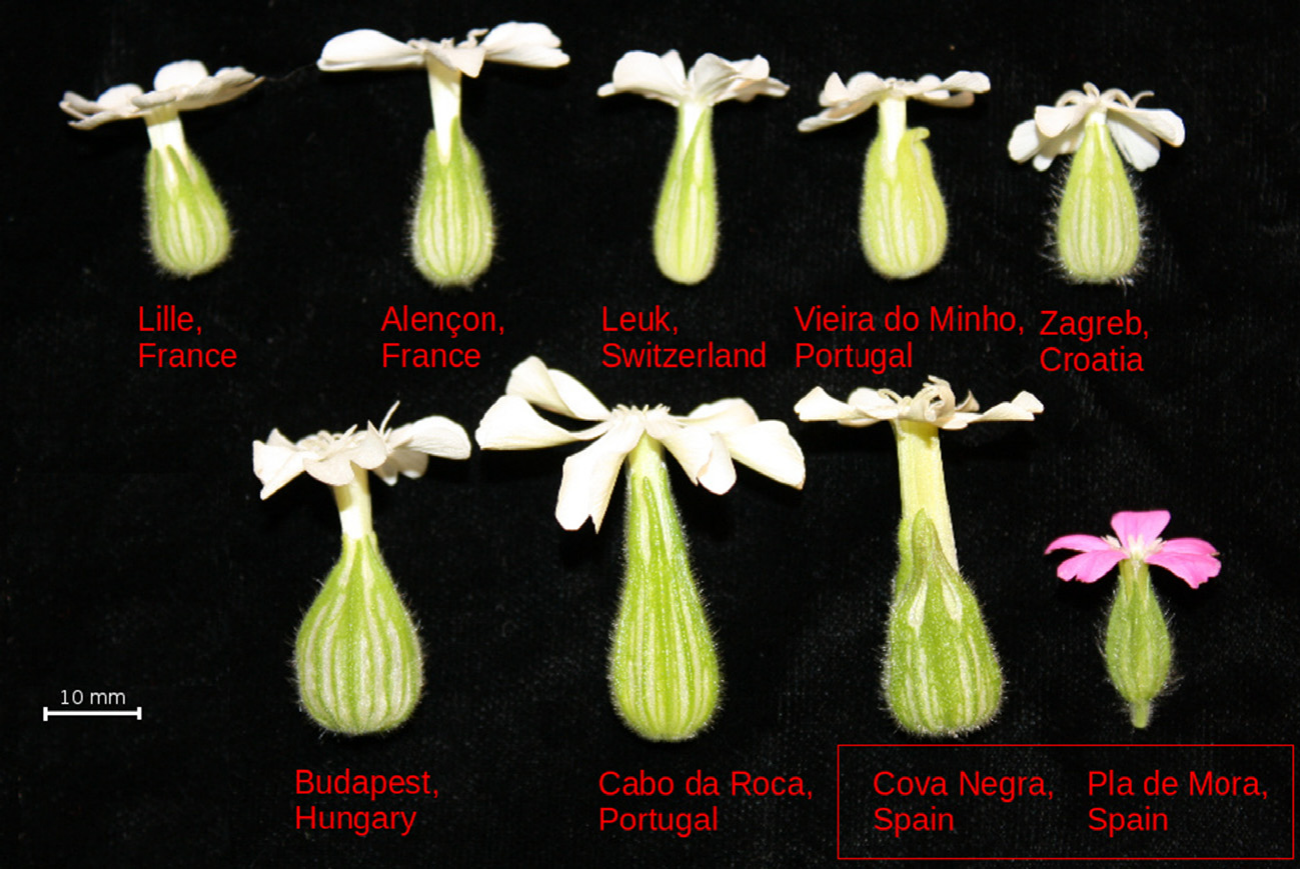
Typical flowers from female plants from each population: white flowers – *S. latifolia*, pink flower – *S. diclinis*. The red box around the labels from Spain indicates sympatry between these populations. All other populations of *S. latifolia* are allopatric with respect to *S. diclinis*.

*Silene latifolia* seed was collected from eight populations throughout its natural European range (Fig.1). Seed from *S. diclinis* was collected from plants growing in Pla de Mora, Spain, nearby the Cova Negra population of *S. latifolia* (CN). The *S. latifolia* population from Cova Negra is sympatric with *S. diclinis*, while the other seven populations we used are allopatric to *S. diclinis*. We will refer to these as the sympatric population and the allopatric populations, respectively.

### Experiment #1: Inter- and intraspecific pollinations and seed set

All plants used in this experiment were from field-collected seeds grown in greenhouses at Indiana University during the spring of 2010 through the summer of 2011. Plants were potted with a 1:1 blend of MetroMix (Scotts-Sierra Horticultural Products, Marysville, OH) and sterilized soil in 13-cm clay pots, with application of 20:20:20 (NPK) and 20:10:30 fertilizers, bi-weekly and monthly, respectively. Artificial light was provided for 14 h each day throughout the year.

Interspecific and intraspecific crosses were made on mothers of both species, using sires from the sympatric population of *S. latifolia*, the seven allopatric populations of *S. latifolia*, and the *S. diclinis* population. Each type of cross was replicated across mothers and sires from either 4 or 5 maternal families per population. Pollen from three flowers was applied by hand along much of the receptive portion of the styles (i.e., the region containing receptive papillae), which runs the entire length of the styles.

Crosses on mothers were performed by simultaneously pollinating sets of flowers within branches, in order to decrease variation in seed set caused by maternal condition or age. For *S. diclinis*, pollinations were made in sets of three using pollen from a *S. diclinis* sire from a different family, a *S. latifolia* sire from sympatry, or a *S. latifolia* sire from allopatry. Similarly, *S. latifolia* mothers were hand-pollinated by a *S. latifolia* sire from their own population but a different maternal family, a sire from the sympatric population of *S. latifolia*, or a sire of *S. diclinis*. At the time of crossing, a flower from each mother was collected from the same branches used for crosses and the number of ovules was counted. This ovule count provided the denominator in our estimate of seed set for each cross, as ovule number is relatively consistent among flowers that open at the same time. Fruits were collected when ripe (but before the top of the fruit was fully opened to disperse the seeds), and the number of seeds counted. Our measure of seed set is the number of seeds in a particular cross, divided by the number of ovules in the flower collected from the same mother. In addition, styles were measured from most mothers used for crosses, in order to look at the relationship between style length and seed set. Style length was measured as the distance from the base of the straightened style to its tip, including the length exerted beyond the corolla opening.

### Experiment #2: Varying interspecific pollen placement

Seeds of *S. latifolia* from five maternal families from each of two populations, the long-styled sympatric population (CN) and a short-styled allopatric population (ZAG), were planted and grown to flowering (as above) in a greenhouse at Indiana University in 2013. We varied the placement of *S. diclinis* pollen on two open flowers per female (1 to 3 females/family for a total of *N* = 22 females), with pollen being placed at the tip of the style on one flower and at the base of the style on the other flower. Within a female, the two pollinated flowers were located on the same branch and shared a lower node, negating the need to count ovules, as the ovule number of such flowers does not differ (data not shown). Fruits were allowed to ripen, and seeds were collected and counted as above. Style length was measured on a third flower open at the same time on each plant.

### Statistical analyses

Statistics were performed in the *R* statistical computing environment (R Core Team 2013) or using JMP version 11.0. We used a one-way ANOVA followed by Tukey’s HSD post hoc test to compare the proportion of seed set on *S. diclinis* mothers when *S. diclinis*, sympatric *S. latifolia*, or allopatric *S. latifolia* sires from LiL, PO, F3, and LK populations (see Fig.1 for abbreviations) were used. Similarly, this test was applied to *S. latifolia* mothers using *S. diclinis*, sympatric *S. latifolia*, or same-population *S. latifolia* sires.

**Figure 2:**
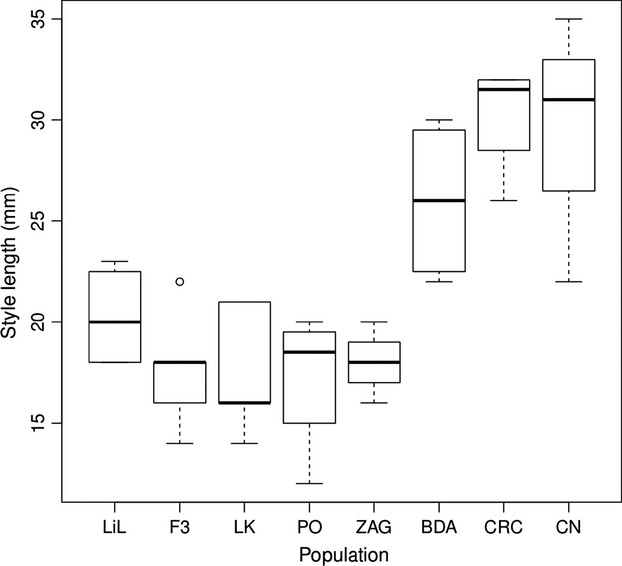
Style lengths of flowers from *S. latifolia* females used in Experiment #1 (in the same order as pictured in Fig.1). LiL – Lille, France; F3 – Alençon, France; LK – Leuk, Switzerland; PO – Vieira do Minho, Portugal; ZAG – Zagreb, Croatia; BDA – Budapest, Hungary; CRC – Cabo da Roca, Portugal; CN – Cova Negra, Spain. The CN population of *S. latifolia* is sympatric with *S. diclinis*; all other populations are allopatric. In comparison, the styles of *S. diclinis* average 9.4 mm in length.

The distribution of proportion seed set on *S. latifolia* mothers appeared to be right-skewed toward higher values; however, a Shapiro–Wilk test of normality failed to reject the hypothesis that the data were drawn from a normal distribution (*N* = 70, *W *=* *0.98, *P *=* *0.53), validating an analysis of variance approach to test for differences. Hence, in order to test whether postmating-prezygotic reproductive isolation of *S. latifolia* from *S. diclinis* is driven by style length vs. sympatry, the seed set of fruits produced by *S. latifolia* mothers was analyzed by fitting an ANCOVA model. To ensure that the ANCOVA model employed appropriately fit the seed set data, Akaike Information Criteria (AIC) and Bayesian Information Criteria (BIC) scores were calculated for a series of reasonable models in which the proportion of seed set per cross was the dependent variable. The model selection procedure included all possible models that included sire species, dam style length, and dam population as factors, as well as all possible interaction terms. Although AIC and BIC scores largely corresponded, the models that minimized these scores differed (data not shown). The optimal BIC model included sire species as a factor, style length of the mother as a covariate, and their interaction, while the optimal AIC model included dam population as well. These two models were nested and so a likelihood-ratio test was applied to select the best-fitting model that did not overfit the data. The larger model, which included dam population as a factor controlling for population-level differences in female fertility, was favored by the likelihood-ratio test (*χ*^2^ = 20.93, df = 7, *P *=* *0.0039); accordingly, this ANCOVA model was used. A *post hoc t*-test was then performed to determine whether the ratio of seed set from crosses with *S. diclinis* sires to the seed set from crosses with same-population *S. latifolia* sires varied significantly between crosses performed with mothers from large- vs. small-flowered populations. The large-flowered population grouping consisted of two populations allopatric to *S. diclinis* (Budapest, Hungary; and Cabo da Roca, Portugal) and the one population sympatric to *S. diclinis* (Cova Negra, Spain), shown in the bottom row of Fig.1. The small-flowered population grouping consisted of five allopatric populations (Lille, France; Alençon, France; Leuk, Switzerland; Vieira do Minho, Portugal; and Zagreb, Croatia), depicted in the top row of Fig.1. Style lengths from typical flowers from female plants from each population are shown in Fig.2.

For Experiment #2, style length was compared with a two-sample *t*-test assuming unequal variances. To compare the number of seeds produced by *S. latifolia* mothers from the two populations (CN vs. ZAG), one t-test was used to compare the means from the base-pollination treatment, and one t-test was used to compare the means from the tip-pollination treatment. For the latter, both a two-sample test assuming unequal variances and a one-sample test (with a null hypothesis of the mean equaling zero) gave the same result, so results from the two-sample test are reported here.

## Results

### Experiment #1

**Figure 3:**
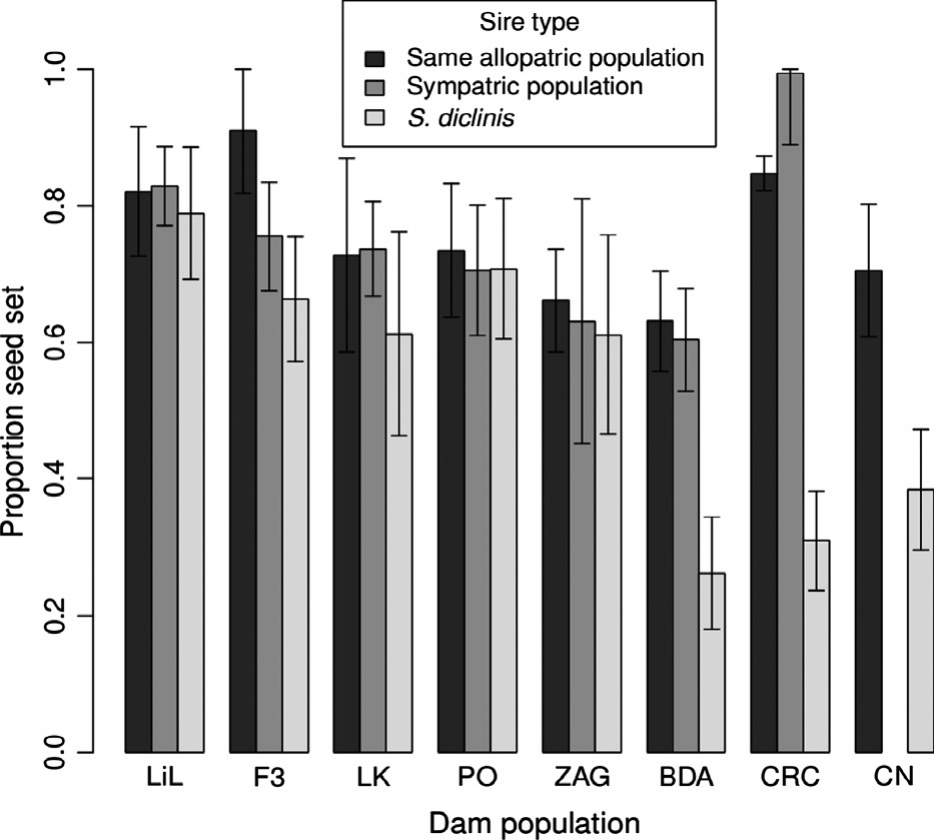
Mean (±1 SE) seed set among *S. latifolia* mothers when fertilized by pollen from their own population (same allopatric population), the sympatric *S. latifolia* population (sympatric population), or *S. diclinis*. Abbreviations for populations of *S. latifolia* are as in Fig.2. The three large-flowered populations are represented by the three rightmost sets of bars.

The styles of *S. diclinis* (mean = 9.4 mm) were shorter than the styles of the shortest-styled population of *S. latifolia*, PO (mean = 17.3 mm; Welch’s two-sample *t*-test: *t*_3.84_ = −4.10, *P *=* *0.008). The proportion of seed set from *S. diclinis* mothers did not differ significantly for the three types of crosses (*F*_2,36_ = 0.17, *P *=* *0.84; means ± SEs: *S. diclinis* sires = 0.69 ± 0.042, sympatric *S. latifolia* sires = 0.66 ± 0.047, and allopatric *S. latifolia* sires = 0.69 ± 0.047). In contrast, the proportion of seed set per fruit from crosses with *S. latifolia* mothers varied significantly depending on the type of cross (*F*_2,103_ = 10.11, *P *<* *0.0001; Fig.3). Tukey’s HSD analysis revealed that interspecific crosses (*S. diclinis* sires) resulted in the setting of a lower percentage of ovules developing into seeds (0.54 ± 0.036) than intraspecific crosses (*S. latifolia* sires) from either the same population (0.74 ± 0.039) or the sympatric population (0.74 ± 0.034), but crosses using either type of *S. latifolia* sire did not differ significantly from each other. We therefore pooled crosses using *S. latifolia* sires for further analysis.

**Figure 4:**
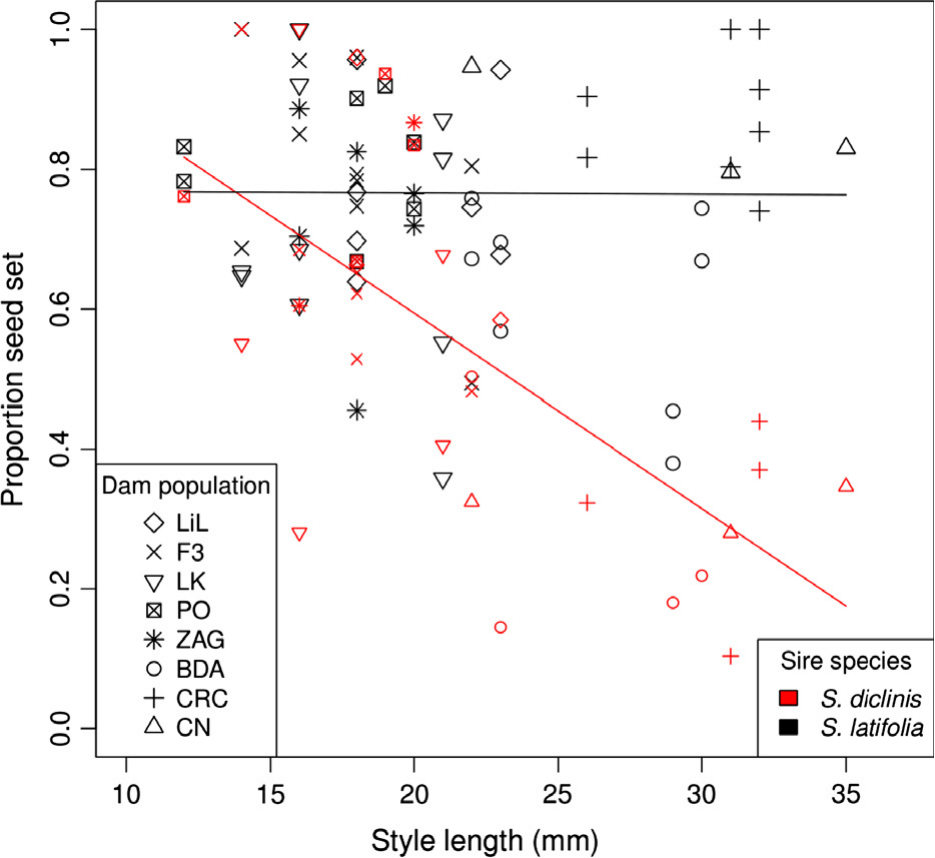
The proportion of seed set per fruit for *S. latifolia* dams decreased with style length when pollinated with *S. diclinis* sires, but not with *S. latifolia* sires. Data points are coded by sire species (*S. latifolia* in black and S*. diclinis* in red) and by the dam’s population (see legend, abbreviations match those in Fig.2). The black and red lines represent the ANCOVA model fit for seed set as a function of style length for *S. latifolia* and *S. diclinis* sired fruits, respectively.

The *S. latifolia* population with the smallest difference in the proportion of seed set between crosses with interspecific vs. intraspecific sires, PO, was also the population with the shortest styles; moreover, the population with the longest styles, CRC, exhibited the largest difference (Figs.2, 3). In the ANCOVA examining a sire species by style length interaction, sire species (*S. latifolia* vs. *S. diclinis*) significantly affected the proportion of seed set per fruit for *S. latifolia* mothers (*F*_1,78_ = 37.12, *P *<* *1 × 10^−7^). Crosses in which *S. diclinis* was the sire resulted in lower seed set. The covariate, style length, also had a significant effect on seed set (*F*_1,78_ = 14.62, *P *=* *0.00026). More importantly, the interaction between sire species and style length was significant (*F*_1,78_ = 22.40, *P *<* *1 × 10^-5^). Seed set decreased with style length when the sire was *S. diclinis*, but not when it was *S. latifolia* (Fig.4). Dam population was also significant, although less so than the other factors in the model (*F*_7,78_ = 2.92, *P *=* *0.009).

Following up on the significant population effect, a post hoc analysis revealed that the ratio of seeds produced by interspecific vs. same-population-intraspecific sires was significantly larger for *S. latifolia* mothers from small-flowered populations as compared to those from large-flowered populations (0.85 + 0.049 vs. 0.47 + 0.091, respectively; Welch’s two-sample *t*-test, *t*_16_ = −3.86, *P *=* *0.0007). In other words, the three large-flowered populations showed more of a decrease in seed set following pollination with *S. diclinis* than did the group of five small-flowered populations (Fig.2).

### Experiment #2

Styles from females used in this experiment from the sympatric long-styled population, CN, were significantly longer than those from the allopatric short-styled population, ZAG (means = 28.2 ± 0.92 vs. 16.2 ± 1.79, respectively; *t*_11.7_ = −18.07, *P *<* *0.0001). While ZAG females produced seeds following tip pollinations with *S. diclinis* pollen, 131.4 (± 16.59), tip pollinations using the 11 CN females resulted in the flowers aborting in all cases (i.e., no seeds were produced). In contrast, the number of seeds produced following base pollinations with *S. diclinis* pollen from these same CN females was close to identical to the number produced by females from ZAG: 285.5 (± 31.43) for CN females and 282.5 (± 27.61) for ZAG females. Accordingly, *t-*tests indicated that the base pollinations with *S. diclinis* pollen did not result in a significantly different number of seeds between populations (*t*_20_ = −0.07, *P *=* *0.94), but seed number following tip pollinations was significantly lower in CN compared to ZAG (*t*_10_ = 7.92, *P *<* *0.001).

## Discussion

Reinforcement has previously been suggested as a mechanism that contributed to the lack of hybrids between *S. latifolia* and *S. diclinis* in nature, based on the inability to produce viable hybrid seeds following hand pollinations (Prentice 1978). Moreover, other aspects of this system suggested that reinforcement was possible, and even likely. These include the two species coming into sympatry after evolving in allopatry (Muir et al. 2012), opportunity for limited pollen flow between the two species in sympatry, and postzygotic reproductive isolation in the form of Haldane’s rule (see Introduction). While our experiments were not sufficient to conclude whether or not reinforcement had occurred, they allowed us to determine whether or not a pattern existed that is commonly consistent with reinforcement, that of greater reproductive isolation in sympatry compared to allopatry. We did not find such a pattern.

Our experiments also allowed us to determine whether style length contributed to reproductive isolation between these two species. We found that style length conferred premating-postzygotic isolation. Our results are consistent with previous studies showing that style-length differences can confer reproductive isolation by impeding gene flow from short-styled species to long-styled species (Buchholz et al. 1935; Grant 1966; Smith 1970; Levin 1978; Whalen 1978; Williams and Rouse 1988; Carney and Arnold 1997; Diaz and Macnair 1999; Tiffin et al. 2001; Kay and Schemske 2008; Lee et al. 2008; Field et al. 2010; Montgomery et al. 2010).

We found that the isolation occurred whenever the flowers of *S. latifolia* were large and contained long styles that *S. diclinis* pollen tubes could not traverse. Such long-styled, large flowers occurred in two of our seven allopatric populations. In fact, we found that styles of *S. latifolia* were longest in one of these allopatric populations, CRC, rather than the sympatric population, CN. Moreover, the population from Hungary (BDA) also had relatively long styles, and this population is geographically distant from the area of sympatry, with many populations of short-styled plants in between. The existence of geographically dispersed allopatric populations with long styles supports the premise that pressures other than the presence of a closely related congener can lead to large flowers in *S. latifolia*. In other words, the presence of *S. diclinis* is not necessary for the evolution of long styles and large flowers in *S. latifolia*, although we cannot completely rule out a possible contribution. Nevertheless, previous findings indicate that flower size is highly labile in *S. latifolia* and responds to a variety of selection pressures, including its seed-predating main pollinator and water availability (e.g., Wright and Meagher 2004; Herlihy and Delph 2009; Burkhardt et al. 2012; Delph and Herlihy 2012). By comparing the two populations we used from Portugal, which vary markedly in flower size, this lability can be seen (Fig.1). Had we not included the two long-styled allopatric populations, the data would have much more congruent with the hypothesis that the long styles exhibited by *S. latifolia* in sympatry evolved specifically to reduce hybridization. Our findings highlight the importance of including a large number of allopatric populations in the study of reinforcement in order to capture the relevant variation in the trait of interest, especially when the trait is quantitative.

We found that the pattern of asymmetric postmating-prezygotic reproductive isolation between *S. latifolia* and the closely related *S. diclinis* was related to among-population variation in style length. Ovules of the relatively short-styled *S. diclinis* flowers were fertilized at the same rate by all sires, even those of *S. latifolia*. In contrast, when long-styled populations of *S. latifolia* were used as the mother, *S. diclinis* pollen was unable to achieve full fertilization of the ovules. This effect was even more extreme when interspecific pollen was placed only at the tip of the styles of flowers from a population with relatively long styles, as no seeds were formed. Furthermore, by effectively shortening styles via pollinations at the base of the style, we showed that pollen from *S. diclinis* was just as capable of fertilizing ovules in long-styled flowers as it was in relatively short-styled flowers when it did not have to travel down the length of the style. This result rules out alternative factors other than style length as the cause of the incompatibility. Overall, our results clearly show that the longer styles of *S. latifolia* are a mechanical postmating-prezygotic barrier to gene flow, leading to asymmetry in prezygotic isolation with *S. diclinis*.
